# Biosensors for point-of-care testing and personalized monitoring of gastrointestinal microbiota

**DOI:** 10.3389/fmicb.2023.1114707

**Published:** 2023-05-05

**Authors:** Lightson Ngashangva, Santanu Chattopadhyay

**Affiliations:** ^1^Transdisciplinary Biology, Rajiv Gandhi Centre for Biotechnology (RGCB), Thiruvananthapuram, Kerala, India; ^2^Pathogen Biology, Rajiv Gandhi Centre for Biotechnology (RGCB), Thiruvananthapuram, Kerala, India

**Keywords:** probiotics, biosensors, point-of-care devices, personalized healthcare, gastrointestinal microbiota

## Abstract

The gastrointestinal (GI) microbiota is essential in maintaining human health. Alteration of the GI microbiota or gut microbiota (GM) from homeostasis (i.e., dysbiosis) is associated with several communicable and non-communicable diseases. Thus, it is crucial to constantly monitor the GM composition and host–microbe interactions in the GI tract since they could provide vital health information and indicate possible predispositions to various diseases. Pathogens in the GI tract must be detected early to prevent dysbiosis and related diseases. Similarly, the consumed beneficial microbial strains (i.e., probiotics) also require real-time monitoring to quantify the actual number of their colony-forming units within the GI tract. Unfortunately, due to the inherent limitations associated with the conventional methods, routine monitoring of one’s GM health is not attainable till date. In this context, miniaturized diagnostic devices such as biosensors could provide alternative and rapid detection methods by offering robust, affordable, portable, convenient, and reliable technology. Though biosensors for GM are still at a relatively preliminary stage, they can potentially transform clinical diagnosis in the near future. In this mini-review, we have discussed the significance and recent advancements of biosensors in monitoring GM. Finally, the progresses on future biosensing techniques such as lab-on-chip, smart materials, ingestible capsules, wearable devices, and fusion of machine learning/artificial intelligence (ML/AI) have also been highlighted.

## 1. Introduction

Trillions of microorganisms (bacteria, archaea, viruses, fungi, and protozoa) colonize in human gastrointestinal tract forming GM, which has a significant role in maintaining gut homeostasis (Schmidt et al., 2018). Within GM, these microbes co-evolve with the host and maintain a mutually beneficial relationship (Shreiner et al., 2015; Cani, 2018; Durack and Lynch, 2019). However, such symbiotic relationships are at constant threat of disruption, leading to the overgrowth of a particular pathobiont. The resulting gut dysbiosis is known to be linked to several communicable and non-communicable diseases (Kho and Lal, 2018; Daliri et al., 2020). Besides, the human gut may also get infected by enteric pathogens leading to serious health concerns, including mortality. The resident microbiota and the invading pathogens generate many metabolic products, which positively or negatively impact human health (Federici, 2019; de Vos et al., 2022).

Since the resident GM influences many physiological activities of the host, like immune response, nutrition, and metabolism, it is crucial to assess and monitor the composition of GM in real-time. In order to identify and determine the GI microflora, different approaches such as biochemical (culture-dependent) and molecular (culture-independent) techniques have been explored (Jian et al., 2020). Furthermore, many advanced high-throughput bio-analytical equipment is available to detect various microbes and their metabolites. Though such equipment offers highly sensitive, selective, and high throughput results, they are expensive, time-consuming, and require technical expertise to operate and interpret the results. Therefore, such instruments cannot be used by everyone and anywhere (Montes-Cebrián et al., 2018).

Since the inception of glucose biosensors such as glucometer, different biosensors have been developed for various applications. With advanced biomedical engineering and biotechnology, nanotechnology, and microtechnology, modern biosensors are extremely useful in healthcare. Recently, several biosensors have been developed to detect pathogens in environmental samples (Ngashangva et al., 2022). In addition, integrating biosensors with inter-disciplinary research fields and advanced information technology, such as wireless and machine learning, is expanding rapidly (Cui et al., 2020). In this mini-review, we briefly discuss the potential of using biosensors as Point-of-Care (POC) and personalized devices for monitoring the pathogenic and beneficial microbes in GM.

### 1.1. GM in health and disease

Microbiota is the consortium of all microbial members colonized in a particular niche. Different human body niches carry different microbiota with distinct qualitative and quantitative compositions. In the human body, the GM is composed of the highest number of microbes of different kingdoms, including members of protozoa, archaea, eukaryotes, viruses, and bacteria (Barko et al., 2018). It is interesting to note that the composition and physiology of the GM that influence human health are also highly dependent on human lifestyle (e.g., diet, alcohol consumption, smoking, medication, stress, and sleep) and the surrounding environment. GM contributes to maintaining human health by preventing pathogenic infections (by occupying the gut space and modulating immunity) and by contributing to the nutrition and metabolism of the host (Thursby and Juge, 2017; Schmidt et al., 2018). However, the homeostasis in the GM may get disrupted due to alterations in either lifestyle or environment, leading to dysbiosis and associated complications (Bajaj et al., 2014). Dysbiosis in GM, inadequate nutrition, exposure to enteric pathogens due to improper hygiene early in life may result in environmental enteropathy, which impairs the immune, metabolic, and neuroendocrine physiology that may lead to the long-term cognitive deficit and poor vaccine efficacy (Watanabe and Petri, 2016; Hajela et al., 2020; Singhvi et al., 2020). In addition, dysbiosis may produce microbe-derived metabolites that are detrimental to the host and may result in diseases like non-alcoholic fatty liver disease (NAFLD) (Yuan et al., 2019). Maintaining a high diversity in GM is essential for gut homeostasis. The beneficial microbes like *Lactobacillus plantarum* and the bacteria-derived short-chain fatty acids such as butyrate are crucial in maintaining epithelial integrity (Karczewski et al., 2010).

Moreover, recent studies found that the abundance of two bacterial species, *Faecalibacterium prausnitzii* and *Roseburia hominis*, in the colon is significantly lower for the patients with ulcerative colitis than for the controls (Machiels et al., 2014). Likewise, a lower abundance of *Bifidobacterium* in the gut is related to peptic ulcer and gastric cancer (Devi et al., 2021). In contrast, *Fusobacterium nucleatum* colonization is positively associated with colon cancer (Kostic et al., 2012; Repass et al., 2018). Besides, by incorporating metagenomic sequencing technologies, large data comparison and analysis of the microbial communities have yielded the link between microbiome alteration with human diseases such as cancer (Yu et al., 2017; Thomas et al., 2019; Wirbel et al., 2019), type II diabetes (Wang et al., 2012), cirrhosis (Oh et al., 2020). For detailed perspectives on the inter-relationship between gut microbiota and human health, the authors refer to Zheng et al. (2018), Durack and Lynch (2019), and Gibbons et al. (2022).

Understanding the interplay among GM, gut microbial metabolites, and the host in homeostasis and disease is one of the top challenges in modern science. Modern research on GM is now rapidly moving from relative to quantitative approaches, potentially revealing more information relevant to human health.

## 2. Biosensors as point-of-care diagnostics for GM

Microbial colonization in the human body can be identified using high-throughput diagnostic equipment that detects microbial nucleic acids, microbial proteins, and human antibody titers against specific antigens. Molecular techniques such as Enzyme-Linked Immuno-Sorbent Assay (ELISA) (Yilmaz et al., 2006), polymerase chain reaction (PCR) (Kim et al., 2020), fluorescent *in situ* hybridization (FISH) (Frickmann et al., 2017), etc., have been utilized to analyze human GI microflora. Additionally, microarray techniques such as DNA (Rivas et al., 2018), oligonucleotide (Wang et al., 2002), phylogenetic-microarray (Rigsbee et al., 2011), etc., are explored to meet the demand of simultaneous detection and quantifications of thousands of genes or target sequences within shorter period.

However, such classical instruments have several limitations, like high expense, less portability, the requirement of highly trained personnel, and lengthier procedure. In contrast, miniaturized diagnostic devices such as biosensors are now extensively explored in healthcare monitoring due to their easy operation and portability (Kim et al., 2019).

A biosensor is a device that yields a quantifiable and processable signal corresponding to the concentration of the target analyte. It usually integrates biological sensing elements or bioreceptors or biorecognition element (such as antibodies/enzymes/cell/nucleic acid/aptamer, etc.), transducers of the physicochemical signals (semi-conducting materials/nanomaterials, etc.), and digital displays (along with signal amplifier; Perumal and Hashim, 2014). Compared to classical bioanalytical instruments, biosensors may be more affordable, portable, user-friendly, rapid, among others. Additionally, patients can easily use them for routine health monitoring at the POC—diagnostic testing at or near-the-patient—or point-of-need (PON)—broader spectrum including on-site testing of environment, food samples, etc. Personalized healthcare may be achieved as individuals/patients can monitor their health or the efficacy of the treatment by biosensors.

**Figure 1:**
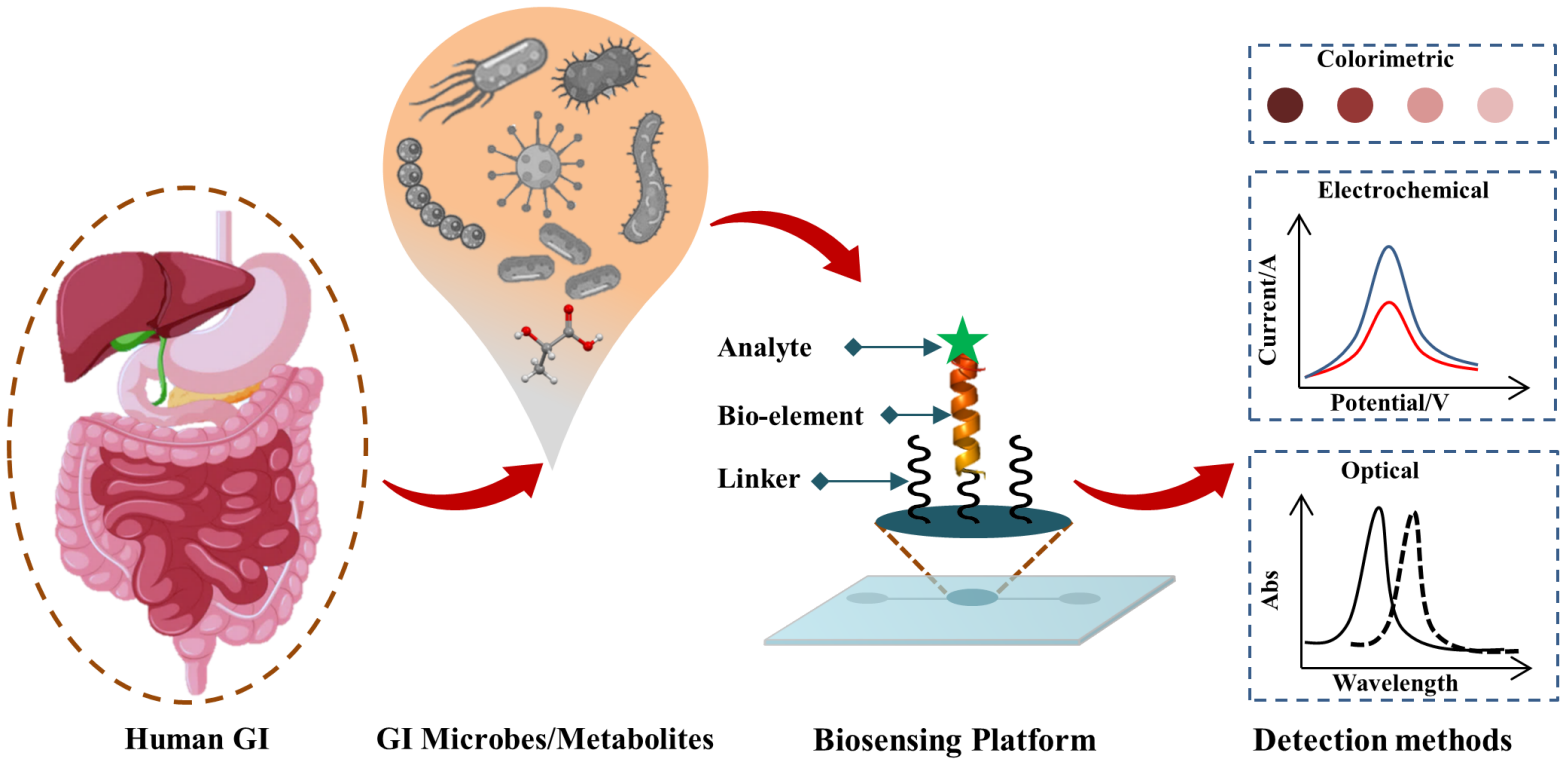
Schematic representation of biosensing techniques for GI microbes and gut metabolites. The target analyte (such as GI microbe or gut metabolite of interest) reacts with the corresponding bio-recognition element (bioelement or bioprobe) of the biosensor and such analyte-bioprobe interaction is selectively and sensitively analyzed using different detection methods such as optical, electrochemical, piezoelectric, thermoelectric, field-effect transistor (FET) approaches.

Biosensors as POC testing for pathogenic, beneficial gut microbes, and gut microbial metabolites are rapidly emerging Some of the recently developed biosensors and bioelectronics for GI microbes, and gut microbial metabolites are summarized in Table 1, and a schematic representation of the biosensors for GM is shown in Figure 1.

**Table 1:** Some of the recent development of biosensors and bioelectronics for GI microbes and gut metabolites.

Biosensors	Biorecognition element	Target analyte	LOD (Sample)	Range	Reference
Optical	Oligonucleotide – AuNPs	*Helicobacter pylori*	25 cfu/mL (Feces)	100–1,000 cfu/mL	Fei et al. (2022)
Optical	Cu^2+^ Nanoflowers	*Helicobacter pylori*	50 cfu/mL (Artificial saliva)	0–10^5^ cfu/mL	Wang T. et al. (2021)
Optical	Aptamer-Fe_3_O_4_ super-paramagnetic NPs	*Helicobacter pylori*	1 cfu/mL (Human feces)	10–10^7^ cfu/mL	Wang Z. et al. (2021)
Electrochemical	Nucleic acid (DNA)	*Helicobacter pylori*	12 fM (Dental plague)	6.55 pM–32.8 fM	Chen et al. (2018)
Electrochemical	Nucleic acid (DNA)	*Helicobacter pylori* DNA	~6 pmol	5–20 pmol	Del Pozo et al. (2005)
Electrochemical	Bismuth-immobilized carbon nanotube	*Helicobacter pylori* DNA	0.6 μg/mL (patient’s gastric tissue)	0.72–7.92 μg/mL	Ly et al. (2011)
Optical (Colorimetric)	AuNPs	*Shigella*	10 fg (culture), 5.86 cfu/mL (human fecal)	10 ng–10 fg	Wang et al. (2016)
Optical (Plasmonic)	*E. faecalis* imprinted nanoparticles	*Enterococcus faecalis*	~100 bac/mL (Sea water)	2 × 10^4^–1 × 10^8^ cfu/mL	Erdem et al. (2019)
Piezoelectric	Sulfo-LC-SPDP & MHDA	*Bifidobacterium bifidum* 01356 & *Lactobacillus acidophilus* 01132	10^3^ cfu/mL (Milk)	10^3^–5 × 10^5^ cfu/ml	Szalontai et al. (2012)
Optical (Fluorescence)	DNAzyme-copper nanoclusters	*E. coli* 0157:H7	1.57 cfu/mL (Drinking water, apple juice)	10–1,000 cfu/mL	Zhou et al. (2020)
Electrochemical (Impedance)	OCMCS-Fe_3_O_4_ NPs	*Campylobacter jejuni*	1 × 10^3^ cfu/mL (Stool)	10^3^–10^7^ cfu/mL	Huang et al. (2010)
Electrochemical	Molecularly imprinted polymer	TMAO	1 ppm/mL (Urine)	1–15 ppm	Lakshmi et al. (2021)
Optical (Colorimetric)	PAH@MnO_2_ nanozyme	TMAO	6.7 μM [Blood (rats)]	15.6–500 μM	Chang Y. C. et al. (2021)
Optical (Colorimetric)	Antibody on lateral flow platform	*Salmonella Typhi*	10 cfu/mL (Fecal)	10^1^–10^7^ cfu/mL	Amalina et al. (2021)
		*Salmonella Paratyphi* A	10^2^ cfu/mL (Fecal)		
Optical (Fluorescence)	Engineered *E. coli* Nissle 1917	Nitrate	39 μM [Fecal, colon (mice)]	0–10 mM	Woo et al. (2020)
Piezoelectric	Antibody-AuNPs	*Bifidobacterium bifidum*	2.1 × 10^2^ cfu/ml [Fecal, food (milk)]	10^3^–10^5^ cfu/mL	Hou et al. (2020)
Optical (Fluorescence)	DNA-mediated Au@Ag@silica nanopopcorn	*Lactobacillus Plantarum*	15 cfu/mL (Mice)	10^5^–10^9^ cfu/mL	Gao et al. (2022)
Optical	Aptamer-decorated porous Si NS	*Lactobacillus acidophilus*	10^6^ cells/mL (selection buffer)	10^6^–10^7^ cfu/mL	Urmann et al. (2016)
Electrochemical (Amperometric)	Lyophilized bacterial cell	Lactate	0.012 mM (Milk, buttermilk, kefir)	0.1–1.0 mM	Canbay et al. (2015)
		Pyruvate	0.018 mM		
Optical (Colorimetric)	Aptamer-AuNPs	*Salmonella enteritidis*	10^1^ cfu/mL (Milk)	10^1^–10^12^ cfu/mL	Fang et al. (2014)
Electrochemical (Impedance)	PPy-Co-CPy – aptamer	*Salmonella typhimurium*	3 cfu/mL (Apple juice)	10^2^–10^8^ cfu/mL	Sheikhzadeh et al. (2016)
Optical (FOLSPR)	Aptamer	*Salmonella typhimurium*	128 cfu/mL (Chicken)	5 × 10^2^–1 × 10^8^ cfu/mL	Xu et al. (2018)

AuNP, Gold nanoparticle; NP, Nanoparticle; LOD, limit of detection; bac/mL, bacteria/mL; cfu, colony forming unit; TMAO, Trimethylamine N-oxide; sulfo-LC-SPDP, sulfosuccinimidyl 6-[3-(2-pyridyldithio)propionamido] hexanoate; MHDA, 16-mercapto-hexadecanoic acid; PAH@MnO_2_, Polyallylamine hydrochloride-capped manganese dioxide; OCMCS-Fe_3_O_4_ NP, O-carboxymethyl chitosan surface modified Fe_3_O_4_ nanoparticle; PPy-Co-CPy, poly[pyrrole-co-3-carboxyl-pyrrole]copolymer; FOLSPR, Fiber-optic localized surface plasmon resonance; Si NS, Silicon nanostructure.

However, as compared to other areas of applications, biosensors for GM remained less progressed, which is partly due to many technical challenges such as: (i) most GI microbes cannot be cultured *in vitro* till date, (ii) it is challenging to isolate and perform standard assays from humans, (iii) challenges that exist to mimic the GI microbial ecosystem in the artificial model, (iv) GM gets altered by many other factors (e.g., diet).

### 2.1. Biosensors for the pathogenic microbes in GM

The biomarkers from GM can be used for assessing various disease progressions, the nature of severity, and the response toward medical treatments. Microbial biomarkers have been proposed for different health problems, such as lung cancer (Liu et al., 2019; Temraz et al., 2019), obesity & diabetes (Zheng et al., 2018; Singer-Englar et al., 2019), and cirrhosis (Bajaj, 2019). Despite difficulties and low success rates, many studies attempted to target gut microbes for diagnostic purposes. Most of these attempts were focused on estimating the metabolites such as glucose, volatiles, endotoxins, etc. (Kassal et al., 2018). Due to the recent attention to the relationship between GM and health, novel microbial biomarkers are now being discovered.

Due to the advancement of synthetic biology and genetic engineering, biosensors such as whole-cell biosensors (WCBs) can detect target analytes directly from complex environments like wastewater or clinical samples. Bacterial biosensors, also called bactosensors, employ whole bacterial cells to determine various pathological biomarkers. Whole-cell bacterial bioreporters are designed to detect toxic chemicals. By integrating with microengineering, genetic circuits could be developed that translate the biochemical signals into quantifiable reporter protein signals (Van Der Meer and Belkin, 2010). Rutter et al. developed an engineered bacterial biosensor using *Caenorhabditis elegans* as a novel model organism to determine isopropyl β-D-1-thiogalactopyranoside (IPTG) in the gut (Rutter et al., 2019). Further advancement in designing the engineered bacterial circuit may help monitor human GM and detect gut inflammation by nitrate sensing (Woo et al., 2020). Sensing the gut metabolites could also provide crucial information on the host–microbe interactions in the gut. With the help of bactosensors, metabolites such as benzoate, lactate, anhydrotetracycline, and bile acids could be detected in human fecal samples. Microbe-associated fecal metabolites has the potential to be used as diagnostics and theranostics explorations (Zúñiga et al., 2022). Another artificial receptor platform called EMeRALD (Engineered Modularized Receptors Activated via Ligand-induced Dimerization) was developed and utilized to detect bile salts (a biomarker of liver dysfunction; Chang H. J. et al., 2021). Biosensors for other essential metabolites, such as trimethylamine-N-oxide (TMAO) (Chang Y. C. et al., 2021; Lakshmi et al., 2021) and indole, have also been developed recently (Wang J. et al., 2021).

Biosensors for detecting gastric pathogen *Helicobacter pylori* have been developed based on different detection methods such as piezoelectric (Su and Li, 2001) and electrochemical (Del Pozo et al., 2005; Ly et al., 2011). Real-time monitoring of the interaction of *H. pylori* with the human gastric mucin was also studied using a resonant mirror-based biosensor (Hirmo et al., 1999). Conversely, *Bifidobacterium bifidum*, proposed to have protective roles against *H. pylori* induced gastric diseases (Alexander et al., 2021; Devi et al., 2021), can be detected using quartz crystal microbalance immunosensor (Hou et al., 2020).

There has been considerable growth in biosensor technology for gut microbiome recently. One of the reasons is the advancement of nanotechnologies/nanoengineering and their use in biosensors technologies (Yadav et al., 2022). Due to their intrinsic properties, nanoparticles are now increasingly used to detect various microbes and their activities in GM (Fuentes-Chust et al., 2021). For example, a plasmonic sensor— based on the molecularly imprinted nanoparticles— has been developed for detecting *Enterococcus faecalis* (Erdem et al., 2019), whereas *Shigella* spp. were detected using gold nanoparticles on lateral flow biosensor (Wang et al., 2016). Another emerging biorecognition element for biosensors is the aptamer biomolecule. Aptamer-based biosensors are significantly explored as they can replace conventional antibody-based diagnostic technologies due to their high specificity and selectivity to bind with the target analyte. A DNA aptamer-based biosensor has recently been developed to study and detect human gut bacterium *Akkermansia muciniphila* (Raber et al., 2021).

### 2.2. Biosensors for beneficial GM

Probiotics are specific strains of beneficial microbial species that provide health benefits to the host upon consumption in sufficient amounts. They are microorganisms with Generally Recognized As Safe (GRAS) status that are critical in preventing diseases like diarrhea, gut inflammation, viral infection, and even colorectal cancer (Uccello et al., 2012; Ghosh, 2018). The probiotics may help to increase the vaccine efficacy in developing countries (Hajela et al., 2020). Nowadays, probiotics are being used even as functional foods. However, probiotics could induce an individualized impact on the gut transcriptome, and the effect may not be universal (Zmora et al., 2018). Recently, probiotics have also initiated personalized therapies (Kort, 2014). Therefore, monitoring quantitative estimation of the probiotics within GM is very important for monitoring GI health and safety, and biosensor could become an instrumental technology.

Although studying the properties of probiotics by using biosensors is not widely used, some attempts have been made. The aptamer (Hemag1P) based biosensors were developed to detect *Lactobacillus acidophilus* with high selectivity and specificity (Urmann et al., 2016). This label-free, simple, and rapid method could distinguish between live and dead bacteria. An aptamer-based electronic biosensor has recently been developed for monitoring the gut bacterium *Roseburia intestinalis* (Xing et al., 2022). The yeasts, also being used as probiotics, have been detected by biosensors (Dacquay and McMillen, 2021). Many essential metabolites from beneficial microbes could be targeted to assess human health. Biosensors have developed for metabolites such as short chain fatty acid butyrate (Bai and Mansell, 2020; Chang Y. C. et al., 2021), lactic acid (Radoi et al., 2010) and indoxyl sulfate (Filik et al., 2016). The immunostimulatory effects usually exerted by the probiotics (e.g., some of the *Lactobacillus* strains) could also be traced by biosensors (Rocha-Ramírez et al., 2017). Importantly, probiotic microbes have been engineered to function as biosensors for detecting autoinducer peptide-I, a quorum-sensing molecule produced by *Staphylococcus* sp. (Lubkowicz et al., 2018). The engineered microbes can increase the complexity, stability, and safety during diagnosis or therapeutics, as observed with smart engineered probiotics (Rottinghaus et al., 2020).

### 2.3. Emerging biosensors for GM

New biosensing concepts and techniques such as *in vivo* wireless and ingestible capsules, microfluidic chips, and internet of things (IoT) have emerged in the recent pasts that are more robust, compact, multiplex, programmable, and reliable. These innovative technologies and smart devices would augment conventional biosensors as efficient and robust POCT devices shortly.

Smart and responsive materials are now being explored to design and develop better and more robust biosensors (Ngashangva et al., 2020). Using such advanced materials, the ingestible capsules were designed to collect microbiome samples from the GI tract. The capsule consisted of 3D-printed acrylic housing, hydrogel, and flexible PDMS membrane (Waimin et al., 2020). This non-invasive sampling technique was validated using *Escherichia coli*. By employing ingestible electronic capsules and a self-powered biosensing system, the crucial metabolite composition of small intestines could also be monitored (De la Paz et al., 2022). Additionally, an ingestible probiotic biosensor has been developed to diagnose GI bleeding in swine. Such ingestible micro-bio-electronic devices have the potential to transform disease management and diagnosis of GI diseases (Mimee et al., 2018). Volatile and gas molecules from a patient’s sample are used as bio-signatures. An electronic nose device based on an array of 13 commercial electro-chemical and optical sensors has been designed to study the microbial volatile metabolites in urine sample of colorectal cancer patients (Westenbrink et al., 2015).

Due to the limitation of the animal model, microfluidic chip such as organ-on-chip has become a promising tool as the physiology and function of tissues or organs can be recapitulated in microfluidic *in vitro* devices. Gut-on-chips are explored to enhance understanding the complex nature of gut microbiota-host interaction (Puschhof et al., 2021; Signore et al., 2021). Another possibility for exploring lab-on-chip technology is multiplex assay and detecting multiple target analytes on a single platform (Shah et al., 2016). The concept of cost-effective, instrument-less, simple, and user-friendly sensing platforms such as microfluidic paper-based analytical devices (μ-PADs) have added benefits to the existing lab-on-chip based biosensor (Takahashi et al., 2022).

As clinical governance is becoming challenging, demanding, and laborious, ML/AI and the internet of medical things must be incorporated with the biosensing concept. Additionally, medical science is transforming from traditional healthcare to digital healthcare monitoring systems that enable improved access to quality healthcare for patients, clinicians, and remote communities. Despite all the advantages, conventional biosensors have limitations such as low specificity, sensitivity, and selectivity compared to other bioanalytical techniques. The overall performance of the biosensor could be improved by integrating with ML/AI approaches. As the individual gut health involves complex interactions between diet, host, microbiota, real-time GM monitoring using ML-based big data collection and analysis with the help of a biosensor could be established (Sosnowski et al., 2020). The ML could also be used to screen the cause-effect relationship between GM dysbiosis and diseases (e.g., cardiovascular diseases; Aryal et al., 2020). ML/AI-based approaches are meta-metabolic network models that are useful to predict the pattern and acquire insights into the synergistic, dysbiotic relationships, and phenotypic outcomes (Lloyd-Price et al., 2019; Cammarota et al., 2020). By understanding such interconnections, development of robust personalized POC biosensors for gut health could be enhanced tremendously.

## 3. Conclusion and future perspective

Since GM is linked to maintaining human physiological processes, monitoring of GM eubiosis is likely to become fundamental in personalized healthcare. Biosensors could be a potential candidate as POC diagnostic devices for personalized monitoring of GM homeostasis. Though biosensors for diagnostics purposes are rapidly advancing, they are still in the preparatory stages for specifically monitoring GM. However, several approaches emerging from diverse fields of science and technology are promising. It is interesting to note that several groups have attempted to develop biosensors for GM by integrating advanced information technologies like ML. It is possible that precise non-invasive monitoring of GM health would be possible in the near future by employing ingestible 3D printed capsules and pairing them with wireless and self-powered wearable electronic devices or even smartphones.

Research on developing biosensors for various gut microbes other than gut bacteria and gut metabolites/biomarkers should be explored to reinforce the personalized biosensor or POCT for GM. Since the results of different studies on gut microbiota are highly variable even in the same disease, methods to develop ultra-sensitive and specificity of biosensors may be focused on in the future, thereby enhancing the early personalized diagnosis and effective medical treatment. Moreover, developing ultra-sensitive biosensors for mental and gut health is an absolute need, as there is a bidirectional relationship between gut microbiota and cognitive behaviors. Furthermore, safety and psychological challenges are associated with continuous monitoring and *in vivo* monitoring devices like ingestible sensors for GM. A minimally invasive approach (such as wearable devices), affordable, and user-friendly (such as μ-PADs) could be more focused in academia and the clinical industries. We believe that the rapid progress of nanotechnology and emerging multidisciplinary approaches would enable early on-site detection of gut metabolites and microbes that empower personalized POC diagnosis with efficient treatment.

## Author contributions

LN designed and drafted the manuscript. SC reviewed and revised the manuscript. All authors contributed to the article and approved the submitted version.

## Funding

This work was supported by intra-mural funding from RGCB, DBT, Govt. of India to LN and SC. It is partially supported by grants to LN (EEQ/2021/000731, SERB-DST), and to SC (MED/2017/46, DBT).

## Conflict of interest

The authors declare that the research was conducted in the absence of any commercial or financial relationships that could be construed as a potential conflict of interest.

## Publisher’s note

All claims expressed in this article are solely those of the authors and do not necessarily represent those of their affiliated organizations, or those of the publisher, the editors and the reviewers. Any product that may be evaluated in this article, or claim that may be made by its manufacturer, is not guaranteed or endorsed by the publisher.
